# Genetic aberrations in glioblastoma multiforme: translocation of chromosome 10 in an O-2A-like cell line

**DOI:** 10.1038/sj.bjc.6690116

**Published:** 1999-02

**Authors:** X Mao, T A Jones, I Tomlinson, A J Rowan, L I Fedorova, A V Zelenin, J-I Mao, N J Gutowski, M Noble, D Sheer

**Affiliations:** Human Cytogenetics Laboratory, Imperial Cancer Research Fund, 44 Lincoln's Inn Fields, London, WC2A 3PX, UK; Cancer Genetics Laboratory, Imperial Cancer Research Fund, 44 Lincoln's Inn Fields, London, WC2A 3PX, UK; Engelhardt Institute of Molecular Biology, Russian Academy of Sciences, Moscow, Russia; Genomics and Technology Development, Genome Therapeutics Corp., 100 Beaver Street, Waltham, MA, 02154, USA; Ludwig Institute for Cancer Research, 91 Riding House Street, London , W1P 8BT, UK

**Keywords:** cytogenetics, FISH, molecular genetics, glioblastoma multiforme

## Abstract

We have examined the genetic aberrations in two near-diploid glioblastoma multiforme cell lines that appear to have arisen from different glial lineages. One cell line, Hu-O-2A/Gb1, expresses antigens and metabolic profiles characteristic of the oligodendrocyte-type-2 astrocyte (0-2A) lineage of the rat central nervous system. This line generates, in vitro, cells with characteristics of 0-2A progenitor cells, oligodendrocytes and astrocytes. The second cell line, IN1434, is derived from an astrocyte or a precursor cell restricted to astrocytic differentiation. In Hu-O-2A/Gb1 the sole homologue of chromosome 10 is disrupted at band 10p11–12.1 by translocation with chromosomes X and 15. The translocation breakpoint is localized between genetic markers D10S2103 and [D10S637, D10S1962, D10S355]. Other aberrations include a 5;14 translocation, deletion of the long and short arms of chromosome 16 and loss of one copy of the *CDKN2* gene. IN1434 cells share some cytogenetic abnormalities with Hu-O-2A/Gb1 cells, despite their apparent derivation from a different biological origin, but also have translocations involving the long and short arms of chromosome 1 and the long arm of chromosome 7, and deletion of chromosome 13 at bands 13q12–21. © 1999 Cancer Research Campaign


*British Journal of Cancer* (1999) **79**(5/6), 724–731
© 1999 Cancer Research Campaign
Article no. bjoc.1998.0116

### Genetic aberrations in glioblastoma multiforme: translocation of chromosome 10 in an O-2A-like cell line

**X Mao[1,*], TA Jones[1], I Tomlinson[2,†], AJ Rowan[2,†], LI Fedorova[3], AV Zelenin[3], J-I Mao[4], NJ Gutowski[5,‡], M Noble[5,§] and D Sheer[1]**

[1]Human Cytogenetics Laboratory and [2]Cancer Genetics Laboratory, Imperial Cancer Research Fund, 44 Lincoln's Inn Fields, London WC2A 3PX, UK; [3]Engelhardt Institute of Molecular Biology, Russian Academy of Sciences, Moscow, Russia, [4]Genomics and Technology Development, Genome Therapeutics Corp., 100 Beaver Street, Waltham, MA 02154, USA; [5]Ludwig Institute for Cancer Research, 91 Riding House Street, London W1P 8BT, UK

**Summary** We have examined the genetic aberrations in two near-diploid glioblastoma multiforme cell lines that appear to have arisen from different glial lineages. One cell line, Hu-O-2A/Gb1, expresses antigens and metabolic profiles characteristic of the oligodendrocyte-type-2 astrocyte (0-2A) lineage of the rat central nervous system. This line generates, in vitro, cells with characteristics of 0-2A progenitor cells, oligodendrocytes and astrocytes. The second cell line, IN1434, is derived from an astrocyte or a precursor cell restricted to astrocytic differentiation. In Hu-O-2A/Gb1 the sole homologue of chromosome 10 is disrupted at band 10p11–12.1 by translocation with chromosomes X and 15. The translocation breakpoint is localized between genetic markers D10S2103 and [D10S637, D10S1962, D10S355]. Other aberrations include a 5;14 translocation, deletion of the long and short arms of chromosome 16 and loss of one copy of the *CDKN2* gene. IN1434 cells share some cytogenetic abnormalities with Hu-O-2A/Gb1 cells, despite their apparent derivation from a different biological origin, but also have translocations involving the long and short arms of chromosome 1 and the long arm of chromosome 7, and deletion of chromosome 13 at bands 13q12–21.

**Keywords:** cytogenetics; FISH; molecular genetics; glioblastoma multiforme

Recent studies have suggested that at least two distinct biological lineages may give rise to glioblastoma multiforme (GBM; Noble et al, 1995). By growing glioma-derived cells in tissue culture conditions previously shown to promote in vivo-like development of glial precursor cells, a human GBM cell line that is unambiguously derived from cells of the human oligodendrocyte-type-2 astrocyte (O-2A) lineage has been isolated. Cells from this line (termed Human O-2A/GlioBlastoma 1, or Hu-O-2A/Gb1) express similar antigens, responsiveness to cytokines and small metabolite profiles (as detected by 1H-nuclear magnetic spectroscopic analysis) to primary O-2A progenitor cells isolated from optic nerves of postnatal rats. In contrast, a second new GBM cell line, designated IN1434, differs from both O-2A progenitor cells and Hu-O-2A/Gb1 cells in most characteristics examined and appears to be derived from an astrocytic lineage.

Consistent genetic aberrations found in GBM include deletion or inactivation of the *CDKN2* gene, amplification and/or overexpression of genes such as *EGFR*, *PDGFR* and *GLI*, as well as loss of heterozygosity (LOH) from chromosomes 13, 17 and 22 (Furnari et al, 1995). A critical step in the development of GBM appears to be LOH of part or an entire homologue of chromosome 10, which occurs almost invariably in GBM but not in lower grade tumors. Mutations have been found in GBM in two novel genes, *PTEN*/*MMAC1* at band 10q23.3 (Li et al, 1997; Steck et al, 1997) and *DMBT1* at 10q25.3–26.1 (Mollenhauer et al, 1997). However, none of the other relevant genes on chromosome 10 have been identified. In order to characterize some of the significant genetic aberrations in GBM, and to determine whether GBMs of different lineages have similar aberrations, we have conducted a detailed genetic analysis of the cell lines Hu-O-2A/Gb1 and IN1434.

### MATERIALS AND METHODS

### Establishment of cell lines

Hu-O-2A/Gb1 was derived from a sporadic temporal GBM in a 59-year-old male; IN1434 was derived from a sporadic frontal GBM in a 70-year-old male. Both tumours were removed surgically before treatment. The specimens were minced using crossed scalpels and incubated in calcium and magnesium Dulbecco's modified Eagle medium–(DMEM–CMF) medium (ICRF) with 2000 units/ml collagenase (Sigma, UK) at 37°C for at least 1 h. For each tumour, parallel cultures were set up with different conditions: DMEM supplemented with 10% fetal calf serum (FCS) and 25 μg $ml^{-1}$ of gentamicin, or serum-free medium (DMEM–BS; Bottenstein and Sato, 1979) mixed in a 50:50 ratio with astrocyte-conditioned medium (ACM). ACM was prepared from growing purified rat cortical astrocytes in DMEM–BS for 48 h (Noble and Murray, 1984; Noble et al, 1984). Cultures were grown in humidified incubators in 5% carbon dioxide at 37°C, and analysed with cell-type specific markers after two passages of in vitro growth and at various subsequent passages up to passage twenty.

*Received 12 December 1997*
*Revised 28 July 1998*
*Accepted 29 July 1998*

*Correspondence to:* D Sheer, Human Cytogenetics Laboratory, Imperial Cancer Research Fund, Lincoln's Inn Fields, London WC2A 3PX, UK

Present addresses: *Section of Molecular Carcinogenesis, Haddow Laboratories, Institute of Cancer Research, 15 Cotswold Road, Sutton, Surrey SM2 5NG, UK; †Molecular and Population Genetics Laboratory, Imperial Cancer Research Fund, 44 Lincoln's Inn Fields, London WC2A 3PX, UK; ‡Neurology Department, Royal Devon and Exeter Hospital (Wonford), Barrack Road, Exeter EX2 5DW, UK; §Huntsman Cancer Institute, Biopolymers Research Building 570, University of Utah Health Sciences Center, Salt Lake City, UT 84112, USA

### Immunohistochemical analysis

Cultures were analysed using monoclonal antibodies A2B5 (Eisenbarth et al, 1979), O1 and O4 (Sommer and Schachner, 1981), anti-galactocerebroside antibody (GalC; Ranscht et al, 1982) and anti-glial fibrillary acidic protein (GFAP; Bignami et al, 1972). A2B5 and O4 have been used previously to characterize rat O-2A progenitor cells (Raff et al, 1983; Barnett et al, 1993). O1 and GalC antibodies specifically label oligodendrocytes, while GFAP is a specific marker of astrocytes.

### Cytogenetic analysis

The cell lines were harvested at passages 14–16 and metaphase chromosomes prepared using standard techniques. Chromosome aberrations were described according to ISCN (Mitelman, 1995). A structural chromosome rearrangement or chromosome gain had to be detected in at least two metaphase cells, and loss of a chromosome in at least three such cells, to be defined as a clonal aberration.

### Fluorescence in situ hybridization

Fluorescence in situ hybridization (FISH) was performed on metaphase spreads according to standard procedures (Senger et al, 1993) using probes shown in Table 1. Chromosome banding was produced by staining with DAPI (diamidino-2-phenylindole-dihydrochloride 0.06 $\mu$g ml$^{-1}$)/propidium iodide (0.5 $\mu$g ml$^{-1}$) dissolved in Cityfluor. Preparations were examined with a Zeiss Axioskop microscope equipped with a cooled charged coupled device (CCD) camera (Photometrics), and digitized images analysed using SmartCapture software (Digital Scientific, Cambridge, UK). At least 50 metaphase spreads and 100 interphase nuclei were analysed for each experiment.

### Microsatellite analysis

Microsatellite analysis of 74 loci derived from the Généthon set (Gyapay et al, 1994) and covering all chromosomes, was performed on the Hu-O-2A/Gb1 cell line only, as control lymphocytes were unavailable for IN1434. A subset of these loci is shown in Table 2. All experiments were conducted ‘blind’. Forty nanograms DNA from cells of passage 5 of Hu-O-2A/Gb1 and control lymphocytes was amplified in 50 $\mu$l of reaction solution containing 2.5 $\mu$l 1 × standard PCR buffer (Promega), 1.5 mM magnesium chloride, 0.4 $\mu$M deoxynucleoside triphosphates (dNTPs), 0.4 $\mu$M of each primer, and 0.5 u *Taq* polymerase. The thermal cycling protocol was 94°C (4 min) × 1, 94°C/55°C (1 min each) × 35, and 72°C (10 min) × 1. Products were electrophoresed for 4 h on 8% non-denaturing polyacrylamide gels, and detected by silver staining using standard methods. Allele loss was scored by eye at informative loci if the intensity of one allelic band in the Hu-O-2A/Gb1 was diminished in comparison with the other allele, allowing for the relative intensity of the alleles in the lymphocyte DNA. Novel alleles were scored as replication errors (RERs).

### Protein truncation test

For mutation analysis of the DNA mismatch repair genes, total mRNA was extracted from cells of passages 5 and 15 of Hu-O-2A/Gb1 using the Quick Prep Micro kit (Pharmacia), and total cDNA made using the First Strand kit (Pharmacia). Pairs of

**Table 1** Probes used for FISH analysis

| Probe[a] | Location | Source (Ref.) |
|---|---|---|
| CHR...B | Specific paints for all chromosomes | Cambio |
| Midi | 1p36.1 | E Volpi |
| pUC1.77 | 1cen | HT Cooke and J Hindley |
| pA3.5 | 3cen | HF Willard |
| Coatasome 5 | 5 paint | Oncor |
| p4A16 | 5+9cen | T Hulsebos |
| YN548 | 5q21 | WF Bodmer |
| Coatasome 7 | 7 paint | Oncor |
| p7E1 | 7cen | EW Jabs |
| LIMK1 | 7q11.23 | X Mao (Mao et al, 1997) |
| 4.4 | 8 cen | A Baldini |
| pM292 | 9 cen | A Baldini |
| *CDKN2* | 9p21.3–22.3 | A Kamb |
| Cathepsin L | 9q13 | X Mao |
| cos50A5 | 9q13 | S Chamberlain |
| AF10[b] | 10p12 | T Chaplin (Chaplin et al, 1995) |
| 14A7[b](D10S2103) | 10p12.1 | J Mao (Ma et al, 1996) |
| JC2139 (D10S637) | 10p11.2 (50 cM) | J Mao (Zheng et al, 1994) |
| 6B2[b] (D10S1962) | 10p11.2 | J Mao (Ma et al, 1996) |
| JC2075 (D10S355) | 10p11.2 (52 cM) | J Mao (Zheng et al, 1994) |
| pA10RR8 | 10 cen | P Devilee |
| sJRH-2b | 10 cen | E Volpi |
| FGF8 | 10q23 | C Dickson |
| EIF5AP1 | 10q23.3 | A Steinkasserer (Steinkasserer et al, 1995) |
| Oligo[c] | 12 cen | E Volpi |
| *GLI/CHOP* | 12q13.3q14.1 | R Anand |
| *CDK4* | 12q14 | R Anand |
| *MDM2* | 12q14.3q15 | R Anand |
| 66G11 | 13 cen | N Jankovsky |
| 127B12[d] | 13q12 | N Jankovsky |
| 104H12 | 13q12 | N Jankovsky |
| 43E12 | 13q13 | N Jankovsky |
| 47A12 | 13q14 | N Jankovsky |
| 97G2 | 13q14 | N Jankovsky |
| 98G5 | 13q21 | N Jankovsky |
| 170D10 | 13q22 | N Jankovsky |
| 87F7 | 13q32 | N Jankovsky |
| 47Q12 | 13q33 | N Jankovsky |
| 44BO6 | 13q34 | N Jankovsky |
| 54CO2 | 13q34 | N Jankovsky |
| 36CO5 | 13q34 | N Jankovsky |
| pLC11A | 11 + 14 cen | HF Willard |
| cos11 | 14q24.2 | D Bennett |
| pTRA | 15 cen | KH Choo |
| pSE16 | 16 cen | HF Willard |
| CN2.3 | 16p13.1–13.3 | H Durbin |
| CMAR | 16q24.1 | H Durbin |
| D20Z1 | 20 cen | Cambio |
| *AP2-g* | 20q13.2 | H Hurst |
| p141/CH22 | 22 cen | HF Willard |
| E289 | 22q11.1 | P Scambler |
| C614 | 22q11.2 | P Scambler |
| N14C6 | 22q11.2 | P Scambler |
| N14A2 | 22q12.1 | P Scambler |
| LIF | 22q12.3 | P Scambler |
| KI831 | 22q12.3 | P Scambler |
| N78F11 | 22q13.1 | P Scambler |
| N119A3 | 22q13.2 | P Scambler |
| N14C3 | 22q13.2–13.3 | P Scambler |
| N66C3 | 22q13.3 | P Scambler |
| Coatasome X | X paint | Oncor |
| DXZ1 | Xcen | Oncor |
| YAC 8B7 | Xq27.1 | R Vatcheva |
| 27D2 | Xq28 | R Vatcheva |

[a]All probes are cosmids except for chromosome paints, centromeres, and probes designated YAC. [b]The exact order of these probes on chromosome 10 is not known. [c]Primers of PRINS. [d]All the probes on chromosome 13 were mapped during the course of this work.

© Cancer Research Campaign 1999

**Table 2** Cytogenetic and genetic maps[a] of selected microsatellite markers used in analysis of Hu-O-2A/Gb1

| Marker | Cytogenetic | Genetic(cM) |
|---|---|---|
| LOH at *APC* gene | | |
| D5S409 | 5q13q23 | 116 |
| D5S421 | 5q13q23 | 129 |
| D5S404 | 5q13q23 | 135 |
| LOH in chromosome 10 | | |
| D10S189 | 10p13 | 18 |
| D10S211[b] | 10p11.2 | 48 |
| D10S197 | 10p11 | 53 |
| D10S220 | 10q11q21 | 77 |
| D10S210 | 10q21q22 | 95 |
| D10S201 | 10q22 | 116 |
| D10S192 | 10q23q24 | 142 |
| D10S190 | 10q25q26 | 161 |
| D10S186 | 10q26qter | 180 |
| LOH at *MSH2* gene | | |
| D2S119 | 2p16 | 75 |
| D2S391 | 2p16 | 81 |
| D2S288 | 2p16 | 82 |
| D2S123 | 2p16 | 85 |
| LOH at *MLH1* gene | | |
| D3S1561 | 3p23p21 | 59 |
| D3S1277 | 3p23p21 | 60 |
| D3S1611 | 3p23p21 | 60 |
| D3S1612 | 3p23p21 | 60 |
| RER status | | |
| D1S216 | 1p21 | 123 |
| 6D6S434 | 6q21q23 | 113 |
| D8S255 | 8p11 | 69 |
| D11S29 | 11q23 | ? |
| D11S904 | 11p14p13 | 40 |
| D11S1313 | 11p12 | 68 |
| D11S901 | 11q12q13 | 94 |
| D11S968 | 11q22 | 165 |
| D17S787 | 17q24 | 83 |
| D20S100 | 20q13 | 95 |

[a]Mapping information from Whitehead Institute/MIT and Généthon. [b]Mapping from Trybus et al, 1996. LOH, loss of heterozygosity; RER, replication error.

oligonucleotides were used to amplify specifically cDNA from the *hMSH2*, *hMLH1*, *hPMS2* and *GTBP* genes in two parts and the *hPMS1* gene in three parts (Liu et al, 1996). For the *APC* gene, genomic DNA samples from passages 5 and 15 of Hu-O-2A/Gb1 and passage 16 of IN1434 were used as templates. Exon 15, which comprises the greatest part of the coding region of the *APC* gene, was amplified specifically in four parts. The protein truncation test (PTT) was performed using standard procedures (van der Luijt et al, 1997). Protein products were visualized by eye on the autoradiograph and their lengths compared with molecular weight markers and samples of known wild-type genotypes.

### RESULTS

### Immunohistochemical phenotypes

Phenotypes were stable during the entire period tested, over 20 passages. Hu-O-2A/Gb1 cells grew in both sets of culture conditions. In medium with FCS, approximately 80% of cells were positive for the astrocytic marker GFAP, but not for any of the other markers tested. When the cells were grown in serum-free medium with ACM, approximately 30–40% of cells were GFAP-positive alone and up to 60% were O4-positive. Approximately 20–25% of the O4-positive cells were also O1- and GalC-positive. Approximately 1% of cells were A2B5-positive alone while 1–2% were both GFAP- and A2B5-positive. Thus, the Hu-O-2A/Gb1 cultures contained the cell types that together comprise the O-2A lineage.

IN1434 cells grew only in culture medium with FCS. Cells expressed GFAP but did not react with A2B5, 04, 01 or GalC antibodies, indicating that they were committed solely to astrocytic differentiation. In addition, the metabolite composition of IN1434 cells did not resemble that of O-2A progenitor cells, as analysed by proton nuclear magnetic resonance ($^{1}$H-NMR) spectroscopy (M Noble et al, unpublished observations). Therefore, all analyses conducted thus far are most consistent with the view that Hu-O-2A/Gb1 cells and IN1434 cells are derived from different glial lineages.

### Genetic aberrations

*Hu-O-2A/Gb1*

Cultures of Hu-O-2A/Gb1 grown in serum-free medium with ACM were used for all subsequent studies. The chromosomes in 50 banded metaphase spreads were analysed and 11 karyotyped. A chromosome number of 42–78, with a mode of 45, was present. Clonal numerical aberrations were loss of chromosomes 6, 10, 13, 15, 16, 19 and 21, and gain of chromosomes 7 and 8. Every cell had lost one homologue of chromosome 10, and the remaining homologue had additional material on the short arm. Other structural aberrations include 14p+, del(5)(p15), and several markers (Figure 1). Seventy-eight per cent of cells showed trisomy 7 and 22% showed tetrasomy 7. FISH and microsatellite analysis were then used to characterize these aberrations in detail (see below). Each of these approaches gave results that were consistent with each other and with the cytogenetic analysis.

*Translocation of chromosome 10*

Nine microsatellite loci mapping to chromosome 10 (Table 2) showed allele loss along the entire chromosome (Figure 2 A–C). The sole chromosome 10 was translocated at band 10p11–12 to chromosomes 15 and X (Figure 2 D–G, Figure 3). FISH showed that probes AF10 and 14A7 (at 10p12) were translocated to a derivative X chromosome at band Xq27–28, while probes 6B2, JC2139, and JC2075 (at 10p11.23), and pA10RR8 (10cen), sJRH-2b (10cen), FGF8 (10q23) and EIF5AP1 (10q23.3) remained on the derivative chromosome 10. The region of chromosome 15 from band 15q11.2–15qter was shown by FISH to translocate to band 10p11–12 of the derivative chromosome 10. Probe pTRA-20 (15cen) was absent from this chromosome. Probes YAC 8B7 (Xq27.1) and 27D2 (Xq28) were not present on the derivative X chromosome or any other chromosome. The X chromosome paint hybridized only to the derivative X chromosome. The region Xq27-qter thus appears to be entirely lost from the genome.

*dic(5;14)*

In addition to an apparently normal homologue of chromosome 5 and a chromosome 5 with a deletion of band 5p15, most cells (92%) contained a dicentric chromosome with material from bands 5pter–5q11–21 translocated onto the short arm of chromosome 14 (Figure 2H). Chromosome 5 material was not detected by FISH on any other chromosome. Probe YN548 (5q21) was not present on this chromosome. No mutation was detected in exon 15 of the APC gene using the PTT (data not shown).

 © Cancer Research Campaign 1999

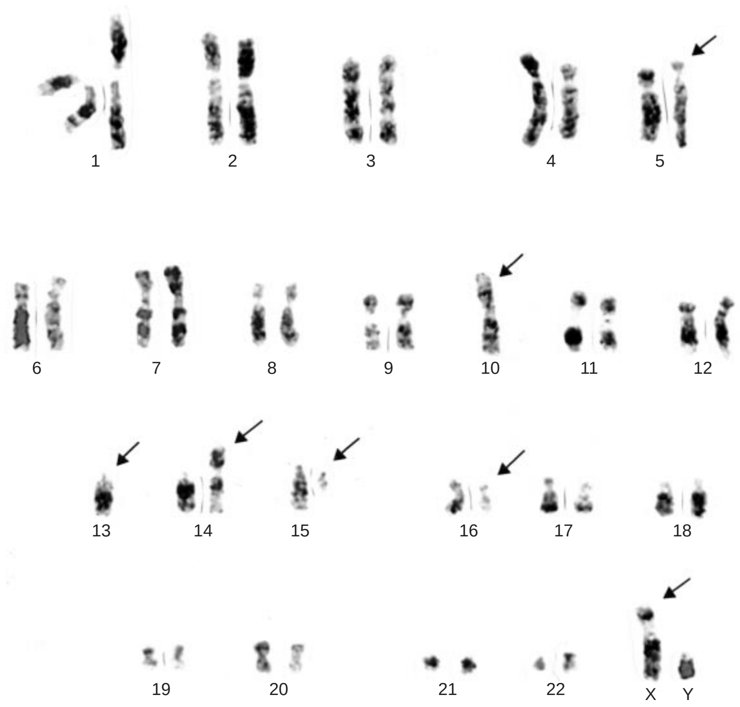

**Figure 1** G-banded karyotype of Hu-O-2A/Gb1. Arrows indicate chromosomes discussed in the text

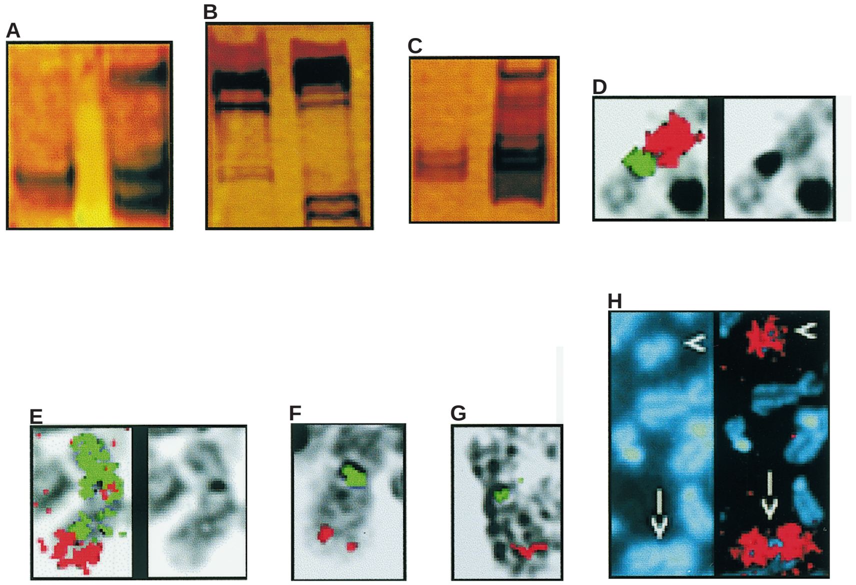

**Figure 2** (**A–C**) Microsatellite analysis of chromosome 10 in Hu-O-2A/Gb1. Left: tumour DNA. Right: normal DNA. (**A**) D10S189 (10pter–p13). (**B**) D10S210 (10q21–22). (**C**) D10S192 (10q23–24). (**D–K**) FISH analysis of Hu-O-2A/Gb1. (**D**) Probes pA10RR8 (10 cen, FITC) and CHR15B (15 paint, Texas red) on the der(10) chromosome. (**E**) Coatasome X (X paint, FITC) and CHR10B (10 paint, Texas red) on the der(X) chromosome. (**F**) Cosmid 14A7 (10p12.1, Texas red) and DXZI (X cen, FITC) on the der(X) chromosome. (**G**) YAC8B7 (Xq27.1, Texas red) and DXZ1 (X cen, FITC) on the der(X) chromosome. (**H**) CHR16B (16 paint, Texas red) on partial metaphase spread stained with DAPI, showing normal and deleted homologues of chromosome 16

### *del(16)(p;q)*

One apparently normal homologue of chromosome 16 was present in every cell, as well as a homologue with both arms deleted (Figure 2I). Probes CN2.3 (16p13.1–13.3) and CMAR (16q24.1) were absent from this chromosome. Microsatellite analysis of eight loci mapping to chromosome bands 16p13.3–q21 showed retention of both alleles at each locus.

### *Other chromosomes*

FISH analysis showed that 57% of cells had lost a copy of the *CDKN2* (p16) gene (9p21–22). FISH analysis using other probes shown in Table 1 failed to detect any abnormalities. Analysis of microsatellite loci (Table 2) in Hu-O-2A/Gb1 cells at passage 5 showed that the tumour was RER–. No allele loss occurred at microsatellite markers close to the *hMSH2* and *hMLH1* genes.

*© Cancer Research Campaign 1999*

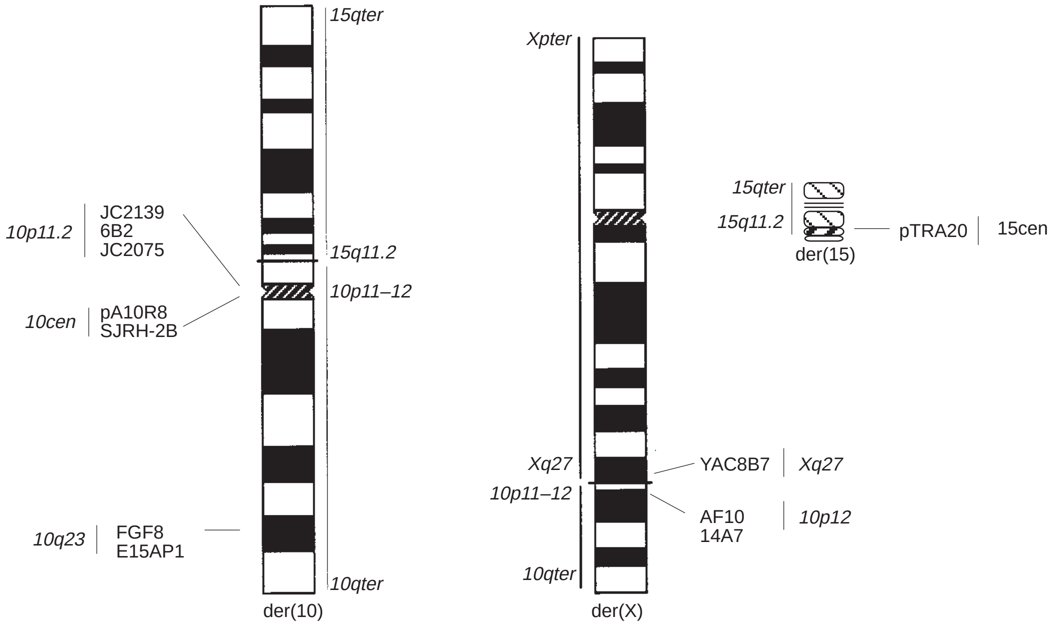

**Figure 3** Diagram of translocations involving chromosome 10 in Hu-O-2A/Gb1. FISH results are shown on the derivative chromosomes

Subsequently, the PTT was used to search for truncating mutations at loci *hMSH2*, *hMLH1*, *hPMS1*, *hPMS2* and *GTBP*. All products amplified from the mRNA of the tumour cells at passage 15 were of wild-type length at these five loci. PTT analysis showed that Hu-O-2A/Gb1 cells did not contain a truncated protein which would indicate a nonsense or frameshift mutation. No abnormalities were detected in the remaining microsatellite loci.

### IN1434

Forty metaphase spreads had a chromosome number of 41–94, with a modal chromosome number of 46 (Figure 4). Eighty-eight per cent of cells were near-diploid cells and 12% were hyperdiploid. Cytogenetic and FISH analysis revealed clonal gains of chromosomes 5, 7, 18, 19 and 20, and clonal losses of chromosomes 6, 8, 10 and 22. FISH and microsatellite analysis were again used to characterize these aberrations in detail, and gave consistent results.

### *t(1;7)*

Each cell had one normal chromosome 1 and a rearranged chromosome 1 with chromosome 7 material translocated to both the long and short arms (Figure 5 A–D). FISH analysis showed that probe p7E1 (7cen) localized to the telomeric region of the long arm of the rearranged chromosome 1. A cosmid probe for the *LIMK1* gene (7q11.23) was found on the telomeric regions of both long and short arms of chromosome 1. This chromosome is thus described as der(1)t(1;7)(p31;q11.23)/(q44;q11).

### *del(13)*

One normal chromosome 13 and a rearranged chromosome 13 were present. Two-colour FISH was performed with 13 cosmid probes covering the entire chromosome 13 (Figure 5 E–G). Cosmids 127B12 (13q12), 43E12 (13q13), 47A12 and 97Q (13q14) were found to be deleted from the rearranged chromosome, indicating an interstitial deletion.

### *Other loci*

FISH analysis with all other probes listed in Table 1 showed normal patterns. No *APC* mutation was detected using the PTT. All other genomic regions tested appeared normal.

### DISCUSSION

We have characterized genetic aberrations in two near-diploid GBM cell lines of different cellular origins. The Hu-O-2A/Gb1 cells grown in serum-free medium with ACM express antigens and have a differentiation potential and $^{1}$H-NMR profile characteristic of the oligodendrocyte-type 2 astrocyte (O-2A) progenitor cell lineage of the rat (Noble et al, 1995). IN1434 cells, in contrast, appear to derive from a lineage committed solely to astrocytic differentiation. Several cytogenetic aberrations in these lines have been noted in other studies of GBM (Mitelman, 1994; Debiec-Rychter et al, 1995). Of particular interest, however, are a translocation of the sole copy of chromosome 10 in Hu-O-2A/Gb1 and a complex rearrangement involving chromosomes 1 and 7 in IN1434.

Previous studies suggest the presence on chromosome 10 of tumour suppressor genes besides *PTEN*/*MMCA1* which are inactivated during progression to GBM (Karlbom et al, 1993; Ichimura et al, 1998). Both cell lines examined here had lost one homologue of chromosome 10. The remaining homologue in Hu-O-2A/Gb1 is translocated at band 10p11.2 to chromosomes 15 and X. The rearrangement is defined as follows: 10pter–[AF10, 14A7] – breakpoint – [JC2139, 6B2, JC2075] – cen. The *AF10* gene which is disrupted in acute leukemia (Chaplin et al, 1995) appears unaffected in Hu-O-2A/Gb1. It is not yet clear whether genetic material has been lost or a gene disrupted by this translocation. If so, the cell line may be useful for cloning a tumour suppressor gene. The same region also shows LOH in prostate cancer (Trybus et al, 1996).

The X;10 translocation in Hu-O-2A/Gb1 also results in deletion of the region Xq27–qter from the genome. Although loss of an

 © Cancer Research Campaign 1999

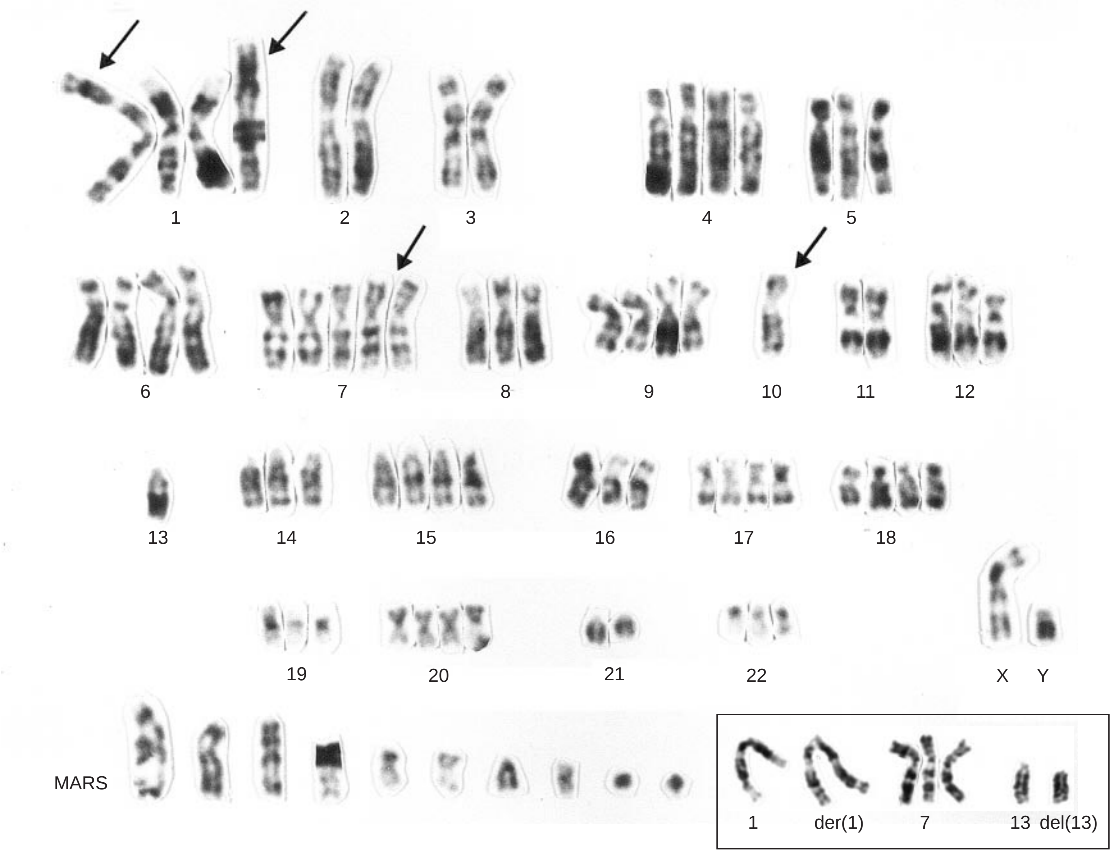

**Figure 4** G-banded karyotype of IN1434. Arrows indicate chromosomes discussed in the text. Inset: partial karyotype showing chromosomes 1, der(1), 7, 13 and del(13)

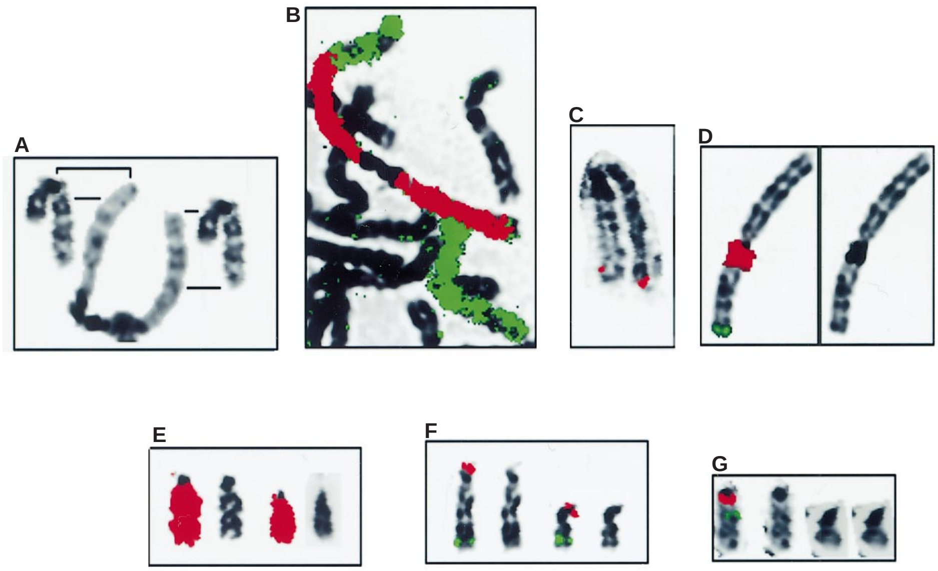

**Figure 5** (**A–D**) FISH analysis of the der(1) in IN1434. (**A**) G-banded der(1) chromosome and chromosome 7, showing regions of chromosome 7 translocated to the der(1). (**B**) CHR1B (1 paint, rhodamine) and CHR7B (7 paint, FITC) showing translocation of chromosome 7 to 1p and 1q. (**C**) Cosmid LIMK1 (7q11.23, rhodamine) hybridizing to both short and long arms of the der(1). (**D**) Probes p7E1 (7 cen, FITC) and pUC1.77 (1 cen, rhodamine) showing 7 cen sequences at the distal long arm region of the der(1). (**E–G**) FISH analysis of del(13) in IN1434. (**E**) CHR13B (13 paint, rhodamine) showing one smaller chromosome 13. (**F**) Cosmids 66G11 (13 cen, rhodamine) and 37Q12 (13q33, FITC) present on both homologues of chromosome 13. (**G**) Cosmids 127B12 (13q12, Texas red) and 14A12 (13q14, FITC) present on the normal chromosome 13 but not on the del(13)

*© Cancer Research Campaign 1999*

entire sex chromosome is common in gliomas, we are unaware of nullisomy for this region of the X chromosome being described previously in GBM.

In Hu-O-2A/Gb1, a region of chromosome 5 from bands 5pter-5q11–21 was translocated to the short arm of chromosome 14, resulting in a dicentric chromosome. Two intact copies of chromosome 5 were also present. Most cases of Turcot's syndrome, which includes gastrointestinal tumours and GBM, result from germ-line mutations of the APC tumour suppressor gene at band 5q21 (Hamilton et al, 1995). APC is therefore a prime candidate tumour suppressor gene for GBM. Microsatellite analysis did not detect allele loss at markers on chromosome 5, and no *APC* mutation was detected by PTT. We have no evidence, therefore, that somatic *APC* mutations were involved in the pathogenesis of Hu-O-2A/Gb1.

We tested Hu-O-2A/Gb1 cells for mutations of five DNA mismatch repair (MMR) genes and for microsatellite instability, since a subset of patients with Turcot's syndrome have germ-line MMR mutations. Neither MMR mutations nor microsatellite instability were detected. Recently other studies failed to detect microsatellite instability in gliomas (Amariglio et al, 1995; Ritland et al, 1995), suggesting that the tumours of Turcot's syndrome may be unrepresentative of sporadic cases.

FISH analysis showed that 57% of Hu-O-2A/Gb1 cells had lost one homologue of a cdk4 inhibitor, CDKN2A, which negatively regulates cell cycle progression. Homozygous deletion, mutation and loss of expression of the *CDKN2* gene are among the most common genetic aberrations in AA and GBM (Kamb et al, 1994; Nobori et al, 1994).

Both Hu-O-2A/Gb1 and IN1434 had trisomy 7 in the majority of cells and tetrasomy 7 in the remaining cells. Trisomy 7 is another common aberration in gliomas. An investigation using comparative genomic hybridization has found that gain of 7q in particular is the most frequent event detected in adult low-grade astrocytomas, suggesting that genes on 7q play an early role in tumour development (Schröck et al, 1996). However, since trisomy 7 is also found in non-neoplastic brain cells, its relevance to glioma development is still debatable (Johansson et al, 1993). No amplification of the *EGFR* gene (7p12) was found by FISH analysis in Hu-O-2A/Gb1 or IN1434 (data not shown). However, high expression of another gene on chromosome 7, *LIMK1* (7q11.2), was observed in paraffin-embedded tumour tissue from which Hu-O-2A/Gb1 was derived (Gutowski et al, in preparation).

Each cell in IN1434 had a complex rearrangement in which chromosome 7 material, including the *LIMK1* gene, was translocated to a derivative chromosome 1 at both the long and short arms. This rearrangement can be described as der(1)t(1;7)(p31;q11.23)/(q44;q11). High expression of *LIMK1* mRNA was also observed in this line (Gutowski et al, in preparation). The expression profiles of *LIMK1* in the two cell lines are consistent with a generally high expression of the gene in normal brain tissue (Proschel et al, 1995).

Cytogenetic and FISH analysis showed that IN1434 had an interstitial deletion of chromosome 13 involving bands 13q12–14. These findings confirm previous studies showing deletion of chromosome 13 in AA and GBM (Kim et al, 1995). The *RB1* gene at band 13q14 is a prime candidate target for the deletion, as it is commonly mutated in astrocytic gliomas. Interestingly, mutation of *RB1* has been found to correlate inversely with mutations of the *CDKN2* gene in gliomas, confirming that perturbation of the cell cycle regulatory pathway that includes *RB1*, *CDKN2* and *CDK4* is a critical step in glioma development (Ueki et al, 1996).

The findings presented here raise intriguing questions concerning the relationship between genetic aberrations and the cellular lineages of tumors. Hu-O2A/Gb1 cells, which by a variety of stringent biological criteria appear to be derived from oligodendrocyte precursors, do not show the cytogenetic aberrations characteristically observed in oligodendrogliomas or oligoastrocytomas such as LOH on 19q and 1p (Kraus, 1995), but rather those seen with great frequencies in other GBMs. Moreover, GBM-associated aberrations also were found in IN1434 cells that appear to be derived from an astrocytic lineage. Answers to these questions will require genetic studies on further gliomas which have been unambiguously assigned to particular lineages.

The extent to which the O-2A lineage contributes to glioma formation itself remains to be determined. However, reports that such tumors frequently express sulfatide and/or GalC (Jennemann et al, 1990; Singh et al 1994), and that some glioma-derived cell lines can be induced to express GalC or mRNA for proteolipid protein (Gillaspy et al, 1993; Kashima et al, 1993), are consistent with the view that the Hu-O-2A/Gb1 cell line is not a unique example of an O-2A tumour.

### ACKNOWLEDGEMENTS

We are grateful to those who kindly provided us with probes used in the study, including Dr Nick Jankovsky for unpublished probes on chromosome 13. XM was a Post-Doctoral Research Fellow of The Royal Society during part of this work. LIF, AVZ and Dr Nick Janovsky were supported by the Russian National Genome Program. LIF was also supported by the Royal Society. NJG was supported by a grant from the Wellcome Trust No. 035404. AVZ was also supported by a special grant of the Russian Federation for leading scientific schools, No. 96-15-97640.

### REFERENCES

Amariglio N, Friedman E, Stiebel OMH, Phelan C, Collins P, Nordenskjold M, Brok-Simoni F and Rechavi G (1995) Analysis of microsatellite repeats in pediatric brain tumors. *Cancer Genet Cytogenet* **84**: 56–59

Barnett SC, Hutchins AM and Noble M (1993) Purification of olfactory nerve ensheathing cells from the olfactory bulb. *Dev Biol* **155**: 337–350

Bignami A, Eng LF, Dahl D and Uyeda CT (1972) Localization of the glial fibrillary acidic protein in astrocytes by immunofluorescence. *Brain Res* **43**: 429–435

Bottenstein JE and Sato GH (1979) Growth of a rat neuroblastoma cell line in serum-free supplemented medium. *Proc Natl Acad Sci USA* **76**: 514–517

Chaplin T, Ayton P, Bernard OA, Saha V, Della Valle V, Hillion J, Gregorini A, Lillington D, Berger R and Young BD (1995) A novel class of zinc finger/leucine zipper genes identified from the molecular cloning of the t(10;11) translocation in acute leukemia. *Blood* **85**: 1435–1441

Debiec-Rychter M, Alwasiak J, Liberski PP, Nedoszytko B, Babinska M, Mrozek K, Imielinski B, Borowska-Lehman J and Limon J and Limon J (1995) Accumulation of chromosomal changes in human glioma progression. A cytogenetic study of 50 cases. *Cancer Genet Cytogenet* **85**: 61–67

Eisenbarth GS, Walsh FS and Nirenberg M (1979) Monoclonal antibodies to a plasma membrane antigen of neurons. *Proc Natl Acad Sci USA* **76**: 4913–4916

Furnari FB, Huang H-JS and Cavenee WK (1995) Genetics and malignant progression in human brain tumors. *Cancer Surveys* **25**: 233–275

Gillaspy GE, Miller RH, Samols D and Goldthwait DA (1993) Antigenic and differentiative heterogeneity among human glioblastomas. *Cancer Lett* **68**: 215–224

Gutowski N, Bevan K, Urenjak J, Bhakoo K, Williams S, Gadian D, Linskey M, Engel U, O'Leary M, Blakemore WF, Mao X, Sheer D and Noble M (1997) Isolation and characterization of a human oligodendrocyte-type-2 astrocyte (O-2A) progenitor cell glioblastoma, (in preparation)

Gyapay G, Morissette J, Vignal A, Dib C, Fizames C, Millasseau P, Marc S, Bernardi G, Lathrop M and Weissenbach J (1994) The 1993–94 Genethon human genetic linkage map. *Nature Genetics* **7**: 246–339

 © Cancer Research Campaign 1999

Hamilton SR, Liu B, Parsons RE, Papadopoulos N, Jen J, Powell SM, Krush AJ, Berk T, Cohen Z, Tetu B, Burger PC, Wood PA, Taqi F, Booker SV, Petersen GH, Offerhaus GJA, Tersmette AC, Giardiello FM, Vogelstein B and Kinzler KW (1995) The molecular basis of Turcots-syndrome. *New Engl J Med* **332**: 839–847

Ichimura K, Schmidt EE, Miyakawa A, Goike HM and Collins VP (1998) Distinct patterns of deletion on 10p and 10q suggest involvement of multiple tumor suppressor genes in the development of astrocytic gliomas of different malignancy grades. *Genes Chrom Cancer* **22**: 9–15

Jennemann R, Rodden A, Bauer BL, Mennel HD and Wiegandt H (1990) Glycosphingolipids of human gliomas. *Cancer Res* **50**: 7444–7449

Johansson B, Heim S, Mandahl N, Mertens F and Mitelman F (1993) Trisomy 7 in nonneoplastic cells. *Genes Chrom Cancer* **6**: 199–205

Kamb A, Gruis NA, Weaver-Feldhaus J, Liu QY, Karshman K, Tavtigian SV, Stockert E, Day III RS, Johnson BM and Skolnick MH (1994) A cell cycle regulator potentially involved in genesis of many tumor types. *Science* **264**: 436–440

Karlbom AE, James CD, Boethius J, Cavenee WK, Collins VP, Nordenskjold M and Larsson C (1993) Loss of heterozygosity in malignant gliomas involves at least three distinct regions on chromosome 10. *Hum Genet* **92**: 169–174

Kashima T, Tiu SN, Merrill JE, Vinters HV, Dawson G and Campagnoni AT (1993) Expression of oligodendrocyte associated genes in cell lines derived from human gliomas and neuroblastomas. *Cancer Res* **53**: 170–175

Kim DH, Mohapatra G, Bollen A, Waldman FM and Feuerstein BG (1995) Chromosomal abnormalities in glioblastoma multiforme tumors and glioma cell lines detected by comparative genomic hybridisation. *Int J Cancer* **60**: 812–819

Kraus JA, Koopmann J, Kaskel P, Maintz D, Brandner S, Schramm J, Louis DN, Wiestler OD and Vondeimling A (1995) Shared allelic losses on chromosomes 1p and 19q suggest a common origin of oligodendroglioma and oligoastrocytoma. *J Neuropath Exp Neurol* **54**: 91–95

Li J, Yen C, Liaw D, Podsypanina K, Bose B, Wang SI, Puc J, Miliaresis C, Rodgers L, McCombie R, Bigner SH, Giovanella BC, Ittmann M, Tycko, Hibshoosh H, Wigler MH and Parsons R (1997) *PTEN*, a putative protein tyrosine phosphatase gene mutated in human brain, breast and prostate cancer. *Science* **275**: 1943–1947

Liu B, Parsons R, Papadopoulos N, Nicolaides NC, Lynch HT, Watson P, Jass JR, Dunlop M, Wyllie A, Peltomaki P, de la Chapelle A, Hamilton SR, Vogelstein B and Kinzler KW (1996) Analysis of mismatch repair genes in hereditary non-polyposis colorectal cancer patients. *Nature Med* **2**: 169–174

Ma NS-F, Zheng C, Benchekroun Y, Deaven LL, Longmire JL, Moir DT and Mao J (1996) Characterization of a flow-sorted human chromosome 10 cosmid library by FISH. *Cytogenet Cell Genet* **74**: 266–271

Mao X, Jones TA, Williamson J, Gutowski NJ, Pröschel C, Noble M and Sheer D (1997) Assignment of the human and mouse LIM-kinase genes (LIMK1; *Limk1*) to chromosome bands 7q11.23 and 5G1, respectively, by in situ hybridisation. *Cytogenet Cell Genet* **74**: 190–191

Mitelman F (1994) *Catalog of Chromosome Aberrations in Cancer* 5th Edn. Wiley-Liss: New York

Mitelman F (ed) (1995) *An International System for Human Cytogenetic Nomenclature*. S. Karger: Basel

Mollenhauer J, Siemann S, Scheurlen W, Korn B, Hayashi Y, Wilgenbus KK, von Deimling A and Poustka A (1997) DMBT1, a new member of the SRCR superfamily, on chromosome 10q25.3–26.1 is deleted in malignant brain tumours. *Nature Genet* **17**: 32–39

Noble M and Murray K (1984) Purified astrocytes promote the division of a bipotential glial progenitor cell. *EMBO J* **3**: 2243–2247

Noble M, Fok-Seang J and Cohen J (1984) Glia are a unique substrate for the in vitro growth of CNS neurons. *J Neurosci* **4**: 1892–1903

Noble M, Gutowski N, Bevan K, Engel U, Linskey M, Urenjak J, Bhakoo K and Williams S (1995) From rodent glial precursor cell to human glial neoplasia in the oligodendrocyte-type-2 astrocyte lineage. *Glia* **15**: 222–230

Nobori T, Miura K, Wu DJ, Lois A, Takabayashi K and Carson DA (1994) Deletions of the cyclin-dependent kinase-4 inhibitor gene in multiple human cancers. *Nature* **368**: 753–756

Proschel C, Blouin MJ, Gutowski NJ, Ludwig R and Noble M (1995) Limk1 is predominantly expressed in neural tissues and phosphorylates serine, threonine and tyrosine residues in vitro. *Oncogene* **11**: 1271–1281

Raff MC, Miller RH and Noble M (1983) A glial progenitor cell that develops in vitro into an astrocyte or an oligodendrocyte depending on the culture medium. *Nature* **303**: 390–396

Ranscht B, Clapshaw PA, Price J, Noble M and Seifert W (1982) Development of oligodendrocytes and Schwann cells studied with a monoclonal antibody against galactocerebroside. *Proc Natl Acad Sci USA* **79**: 2709–2713

Ritland SR, Ganju V and Jenkins RB (1995) Region-specific loss of heterozygosity on chromosome 19 is related to the morphologic type of human glioma. *Genes Chrom Cancer* **12**: 277–287

Senger G, Ragoussis J, Trowsdale J and Sheer D (1993) Fine mapping of the human MHC class II region within chromosome band 6p21 and evalution of probe ordering using interphase fluorescence in situ hybridisation. *Cytogenet Cell Genet* **64**: 49–53

Schröck E, Blume C, Meffert M-C, du Manoir S, Bersch W, Kiessling M, Lozanowa T, Thiel G, Witkowski R, Ried T and Cremer T (1996) Recurrent gain of chromosome arm 7q in low-grade astrocytic tumors studied by comparative genomic hybridisation. *Genes Chrom Cancer* **15**: 199–205

Singh LP, Pearl DK, Franklin TK, Sprin PM, Scheithauer BW, Coons SW, Johnson PC, Pfeiffer SE, Li J, Knott JC and Yates AJ (1994) Neutral glycolipid composition of primary human brain tumors. *Mol Chem Neuropathol* **21**: 241–257

Sommer I and Schachner M (1981) Monoclonal antibodies (O1 and O4) to oligodendrocyte cell surfaces: an immunocytological study in the central nervous system. *Dev Biol* **83**: 311–327

Steck PA, Pershouse MA, Jasser SA, Yung WKA, Lin H, Ligon AH, Langford LA, Baumgard ML, Hattier T, Davis T, Frye C, Hu R, Swedlund B, Teng DHF and Tavtigian SV (1997) Identification of a candidate tumour suppressor gene, *MMAC1*, at chromosome 10q23.3 that is mutated in multiple advanced cancers. *Nature Genet* **15**: 356–362

Steinkasserer A, Jones T, Sheer D, Koettnitz K, Hauber J and Bevec D (1995). The eukaryotic co-factor for the human immunodeficiency virus type 1 (HIV-1) Rev protein, eIF-5A maps to chromosome 10q23.3. Three eIF-5A pseudogenes map to 10q23.3, 17q25 and 19q13.2. *Genomics* **25**: 749–752

Trybus TM, Burgess AC, Wojno KJ, Glover TW and Macoska JA (1996) Distinct areas of allelic loss on chromosomal regions 10p and 10q in human prostate cancer. *Cancer Res* **56**: 226–237

Ueki K, Ono Y, Hensen JW, Efird JT, Vondeimling A and Louis DN (1996) Cdkn2/p16 or Rb alterations occur in the majority of glioblastomas and are inversely correlated. *Cancer Res* **56**: 150–153

Van der Luijt RB, Khan PM, Vasen HFA, Tops CMJ, van Leeuwen-Cornellise ISJ, Wijnen JT, van der Klift HM, Plug RJ, Griffioen G and Fodde R (1997) Molecular analysis of the APC gene in 105 Dutch kindreds with familial adenomatous polyposis: 67 germline mutations identified by DGGE, PTT and southern analysis. *Human Mutation* **9**: 7–16

Zheng C, Dorman TE, Wang MT, Braunschweiger K, Schuster MK, Rothschild CB, Bowden DW, Torrey D, Keith TP, Moir DT and Mao J (1994) Generation of 124 sequence-tagged sites (STSs) and cytogenetic localization of 217 cosmids for human chromosome 10. *Genomics* **22**: 55–67

© Cancer Research Campaign 1999

## References

[PDF_00577] Amariglio N., Friedman E., Mor O., Stiebel H., Phelan C., Collins P., Nordenskjold M., Brok-Simoni F., Rechavi G. (1995). Analysis of microsatellite repeats in pediatric brain tumors.. Cancer Genet Cytogenet.

[PDF_00580] Barnett S. C., Hutchins A. M., Noble M. (1993). Purification of olfactory nerve ensheathing cells from the olfactory bulb.. Dev Biol.

[PDF_00582] Bignami A., Eng L. F., Dahl D., Uyeda C. T. (1972). Localization of the glial fibrillary acidic protein in astrocytes by immunofluorescence.. Brain Res.

[PDF_00584] Bottenstein J. E., Sato G. H. (1979). Growth of a rat neuroblastoma cell line in serum-free supplemented medium.. Proc Natl Acad Sci U S A.

[PDF_00586] Chaplin T., Ayton P., Bernard O. A., Saha V., Della Valle V., Hillion J., Gregorini A., Lillington D., Berger R., Young B. D. (1995). A novel class of zinc finger/leucine zipper genes identified from the molecular cloning of the t(10;11) translocation in acute leukemia.. Blood.

[PDF_00590] Debiec-Rychter M., Alwasiak J., Liberski P. P., Nedoszytko B., Babińska M., Mrózek K., Imieliński B., Borowska-Lehman J., Limon J. (1995). Accumulation of chromosomal changes in human glioma progression. A cytogenetic study of 50 cases.. Cancer Genet Cytogenet.

[PDF_00594] Eisenbarth G. S., Walsh F. S., Nirenberg M. (1979). Monoclonal antibody to a plasma membrane antigen of neurons.. Proc Natl Acad Sci U S A.

[PDF_00596] Furnari F. B., Huang H. J., Cavenee W. K. (1995). Genetics and malignant progression of human brain tumours.. Cancer Surv.

[PDF_00598] Gillaspy G. E., Miller R. H., Samols D., Goldthwait D. A. (1993). Antigenic and differentiative heterogeneity among human glioblastomas.. Cancer Lett.

[PDF_00605] Gyapay G., Morissette J., Vignal A., Dib C., Fizames C., Millasseau P., Marc S., Bernardi G., Lathrop M., Weissenbach J. (1994). The 1993-94 Généthon human genetic linkage map.. Nat Genet.

[PDF_00612] Hamilton S. R., Liu B., Parsons R. E., Papadopoulos N., Jen J., Powell S. M., Krush A. J., Berk T., Cohen Z., Tetu B. (1995). The molecular basis of Turcot's syndrome.. N Engl J Med.

[PDF_00617] Ichimura K., Schmidt E. E., Miyakawa A., Goike H. M., Collins V. P. (1998). Distinct patterns of deletion on 10p and 10q suggest involvement of multiple tumor suppressor genes in the development of astrocytic gliomas of different malignancy grades.. Genes Chromosomes Cancer.

[PDF_00621] Jennemann R., Rodden A., Bauer B. L., Mennel H. D., Wiegandt H. (1990). Glycosphingolipids of human gliomas.. Cancer Res.

[PDF_00623] Johansson B., Heim S., Mandahl N., Mertens F., Mitelman F. (1993). Trisomy 7 in nonneoplastic cells.. Genes Chromosomes Cancer.

[PDF_00625] Kamb A., Gruis N. A., Weaver-Feldhaus J., Liu Q., Harshman K., Tavtigian S. V., Stockert E., Day R. S., Johnson B. E., Skolnick M. H. (1994). A cell cycle regulator potentially involved in genesis of many tumor types.. Science.

[PDF_00629] Karlbom A. E., James C. D., Boethius J., Cavenee W. K., Collins V. P., Nordenskjöld M., Larsson C. (1993). Loss of heterozygosity in malignant gliomas involves at least three distinct regions on chromosome 10.. Hum Genet.

[PDF_00632] Kashima T., Tiu S. N., Merrill J. E., Vinters H. V., Dawson G., Campagnoni A. T. (1993). Expression of oligodendrocyte-associated genes in cell lines derived from human gliomas and neuroblastomas.. Cancer Res.

[PDF_00635] Kim D. H., Mohapatra G., Bollen A., Waldman F. M., Feuerstein B. G. (1995). Chromosomal abnormalities in glioblastoma multiforme tumors and glioma cell lines detected by comparative genomic hybridization.. Int J Cancer.

[PDF_00639] Kraus J. A., Koopmann J., Kaskel P., Maintz D., Brandner S., Schramm J., Louis D. N., Wiestler O. D., von Deimling A. (1995). Shared allelic losses on chromosomes 1p and 19q suggest a common origin of oligodendroglioma and oligoastrocytoma.. J Neuropathol Exp Neurol.

[PDF_00645] Li J., Yen C., Liaw D., Podsypanina K., Bose S., Wang S. I., Puc J., Miliaresis C., Rodgers L., McCombie R. (1997). PTEN, a putative protein tyrosine phosphatase gene mutated in human brain, breast, and prostate cancer.. Science.

[PDF_00648] Liu B., Parsons R., Papadopoulos N., Nicolaides N. C., Lynch H. T., Watson P., Jass J. R., Dunlop M., Wyllie A., Peltomäki P. (1996). Analysis of mismatch repair genes in hereditary non-polyposis colorectal cancer patients.. Nat Med.

[PDF_00652] Ma N. S., Zheng C., Benchekroun Y., Deaven L. L., Longmire J. L., Moir D. T., Mao J. (1996). Characterization of a flow-sorted human chromosome 10 cosmid library by FISH.. Cytogenet Cell Genet.

[PDF_00655] Mao X., Jones T. A., Williamson J., Gutowski N. J., Pröschel C., Noble M., Sheer D. (1996). Assignment of the human and mouse LIM-kinase genes (LIMK1; Limk1) to chromosome bands 7q11.23 and 5G1, respectively, by in situ hybridization.. Cytogenet Cell Genet.

[PDF_00663] Mollenhauer J., Wiemann S., Scheurlen W., Korn B., Hayashi Y., Wilgenbus K. K., von Deimling A., Poustka A. (1997). DMBT1, a new member of the SRCR superfamily, on chromosome 10q25.3-26.1 is deleted in malignant brain tumours.. Nat Genet.

[PDF_00669] Noble M., Fok-Seang J., Cohen J. (1984). Glia are a unique substrate for the in vitro growth of central nervous system neurons.. J Neurosci.

[PDF_00671] Noble M., Gutowski N., Bevan K., Engel U., Linskey M., Urenjak J., Bhakoo K., Williams S. (1995). From rodent glial precursor cell to human glial neoplasia in the oligodendrocyte-type-2 astrocyte lineage.. Glia.

[PDF_00667] Noble M., Murray K. (1984). Purified astrocytes promote the in vitro division of a bipotential glial progenitor cell.. EMBO J.

[PDF_00674] Nobori T., Miura K., Wu D. J., Lois A., Takabayashi K., Carson D. A. (1994). Deletions of the cyclin-dependent kinase-4 inhibitor gene in multiple human cancers.. Nature.

[PDF_00677] Pröschel C., Blouin M. J., Gutowski N. J., Ludwig R., Noble M. (1995). Limk1 is predominantly expressed in neural tissues and phosphorylates serine, threonine and tyrosine residues in vitro.. Oncogene.

[PDF_00680] Raff M. C., Miller R. H., Noble M. (1983). A glial progenitor cell that develops in vitro into an astrocyte or an oligodendrocyte depending on culture medium.. Nature.

[PDF_00683] Ranscht B., Clapshaw P. A., Price J., Noble M., Seifert W. (1982). Development of oligodendrocytes and Schwann cells studied with a monoclonal antibody against galactocerebroside.. Proc Natl Acad Sci U S A.

[PDF_00686] Ritland S. R., Ganju V., Jenkins R. B. (1995). Region-specific loss of heterozygosity on chromosome 19 is related to the morphologic type of human glioma.. Genes Chromosomes Cancer.

[PDF_00693] Schröck E., Blume C., Meffert M. C., du Manoir S., Bersch W., Kiessling M., Lozanowa T., Thiel G., Witkowski R., Ried T. (1996). Recurrent gain of chromosome arm 7q in low-grade astrocytic tumors studied by comparative genomic hybridization.. Genes Chromosomes Cancer.

[PDF_00689] Senger G., Ragoussis J., Trowsdale J., Sheer D. (1993). Fine mapping of the human MHC class II region within chromosome band 6p21 and evaluation of probe ordering using interphase fluorescence in situ hybridization.. Cytogenet Cell Genet.

[PDF_00697] Singh L. P., Pearl D. K., Franklin T. K., Spring P. M., Scheithauer B. W., Coons S. W., Johnson P. C., Pfeiffer S. E., Li J., Knott J. C. (1994). Neutral glycolipid composition of primary human brain tumors.. Mol Chem Neuropathol.

[PDF_00701] Sommer I., Schachner M. (1981). Monoclonal antibodies (O1 to O4) to oligodendrocyte cell surfaces: an immunocytological study in the central nervous system.. Dev Biol.

[PDF_00704] Steck P. A., Pershouse M. A., Jasser S. A., Yung W. K., Lin H., Ligon A. H., Langford L. A., Baumgard M. L., Hattier T., Davis T. (1997). Identification of a candidate tumour suppressor gene, MMAC1, at chromosome 10q23.3 that is mutated in multiple advanced cancers.. Nat Genet.

[PDF_00709] Steinkasserer A., Jones T., Sheer D., Koettnitz K., Hauber J., Bevec D. (1995). The eukaryotic cofactor for the human immunodeficiency virus type 1 (HIV-1) rev protein, eIF-5A, maps to chromosome 17p12-p13: three eIF-5A pseudogenes map to 10q23.3, 17q25, and 19q13.2.. Genomics.

[PDF_00716] Ueki K., Ono Y., Henson J. W., Efird J. T., von Deimling A., Louis D. N. (1996). CDKN2/p16 or RB alterations occur in the majority of glioblastomas and are inversely correlated.. Cancer Res.

[PDF_00724] Zheng C. J., Ma N. S., Dorman T. E., Wang M. T., Braunschweiger K., Soares L., Schuster M. K., Rothschild C. B., Bowden D. W., Torrey D. (1994). Development of 124 sequence-tagged sites and cytogenetic localization of 217 cosmids for human chromosome 10.. Genomics.

[PDF_00719] van der Luijt R. B., Khan P. M., Vasen H. F., Tops C. M., van Leeuwen-Cornelisse I. S., Wijnen J. T., van der Klift H. M., Plug R. J., Griffioen G., Fodde R. (1997). Molecular analysis of the APC gene in 105 Dutch kindreds with familial adenomatous polyposis: 67 germline mutations identified by DGGE, PTT, and southern analysis.. Hum Mutat.

